# Next-generation sequencing of the mitochondrial genome of *semanotus bifasciatus* (coleoptera: Cerambycidae)

**DOI:** 10.1080/23802359.2019.1671245

**Published:** 2019-09-27

**Authors:** Xiongfei Yan, Yonghua Liu, Gang Li

**Affiliations:** College of Life and Sciences, Yulin University, Yulin, China

**Keywords:** Cerambycidae, Juniper Bark Borer, mitogenome, phylogenetic tree

## Abstract

The Juniper Bark Borer *Semanotus bifasciatus* belongs to family Colubridae, and is distributed in north China, Japan and the Korean Peninsula. In this study, the total mitochondrial genome of *S. bifasciatus* was determined using next-generation sequencing. The whole mitogenome is a typical circular DNA molecule of 16,051 bp and contains 13 protein-coding genes, 22 transfer RNA genes, 2 ribosomal RNA genes and one control region, with a base composition of A 40.8%, G 11.0%, T 32.6%, and C 16.6%. Phylogenetic analysis indicated that *Semanotus bifaciatus* was the nearest sister to *Xylotrechus grayii*. The molecular data presented here would be useful for further study of *S. bifasciatus*.

The Juniper Bark Borer, *Semanotus bifasciatus* belongs to the family Cerambycidae and the species is found in north China, Japan and the Korean Peninsula (Wu and Jiang 1986; Iwata et al. 2007). It is one of the most serious stem borers in Cupressaceae trees.

Limited mitochondrial genome sequences have been sequenced from family Cerambycidae, which comprises over 20 000 described species worldwide (Song et al. 2017). Here, we determined the complete mitochondrial genome of *S. bifasciatus*, with the adult individual, collected from *Platycladus orientalis* in Woyunshan Botanical Garden (latitude: 38.189°N, longitude: 109.865°E), Yulin, Shaanxi Province. The specimen was deposited in Herbarium of Animals and Plants of Yulin University with an accession number YLU-Wsb-20190426.

Total was extracted from muscle tissue using the standard phenol-chloroform protocol. The mitochondrial genome sequence was obtained by next-generation sequencing. The short-insert libraries were sequenced using Illumina Hiseq 4000 (Borgstrom et al. 2011). The de novo assembly of the mitochondrial contigs was conducted by SOAPdenovo2.04 (Luo et al. 2012).

The complete mitochondrial genome of *S. bifasciatus* (GenBank Accession Number MN095416; 16, 051 bp) contains 13 protein-coding genes, 22 transfer RNA genes, 2 ribosomal RNA genes and one control region. The whole base composition of the mitochondrial genome is showed as follows: A 40.8%, G 11.0%, T 32.6%, and C 16.6%; with an A + T-rich pattern of the invertebrate mitochondrial genomes (Wang et al. 2013; Li et al. 2016; Song et al. 2017).

Total length of the 13 protein-coding genes is 11, 167 bp, all of which are encoded on the heavy strand except for Nd5, Nd4, Nd4l and Nd1 in the light strand.

A phylogenetic tree (Figure 1) of mitochondrial genomes analyses of 9 species snakes of Cerambycidae and plus three outgroup species, *Diabrotica barberi* (NC022935), *Galeruca daurica* (NC027114) and *Agasicles hygrophila* (KR494279), was constructed based on the NJ method.

**Figure 1.**
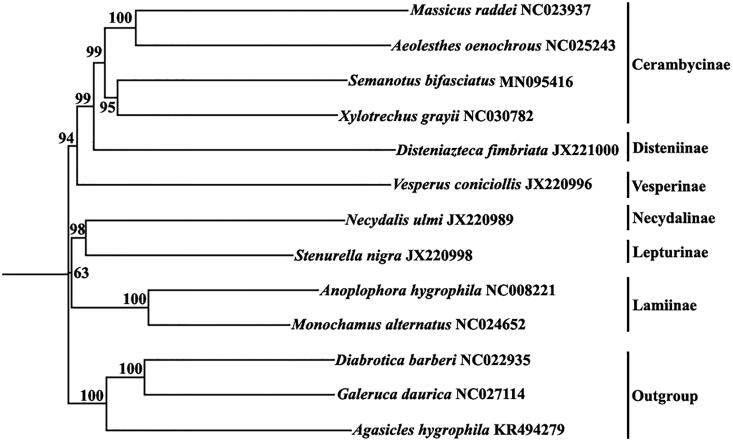
A phylogenetic tree generated using the NJ method based on complete mitochondrial genomes.

Phylogenies, ((Cerambycinae, Disteniinae) Vesperinae) were robustly recovered (Figure 1). Within Cerambycinae, ((*Semanotus*, *Xylotrechus*) (*Massicus*, *Aeolesthes*) were highly supported. *Anoplophora* clustered with *Monochamus*, which was in concordant with Li et al. (2016). *Semanotus bifaciatus* was the nearest sister to *Xylotrechus grayii*.

So far, the species status of *S. bifasciatus* within Cerambycidae is unclear. The complete mitochondrial genome of *S. bifasciatus* we determined would be useful in systematics and population genetics.
